# Accounting for the Material Stock of Nations

**DOI:** 10.1111/jiec.12114

**Published:** 2014-04-10

**Authors:** Tomer Fishman, Heinz Schandl, Hiroki Tanikawa, Paul Walker, Fridolin Krausmann

**Keywords:** construction stocks, industrial ecology, material efficiency, material flow analysis (MFA), social metabolism, stock and flow modeling

## Abstract

National material stock (MS) accounts have been a neglected field of analysis in industrial ecology, possibly because of the difficulty in establishing such accounts. In this research, we propose a novel method to model national MS based on historical material flow data. This enables us to avoid the laborious data work involved with bottom-up accounts for stocks and to arrive at plausible levels of stock accumulation for nations. We apply the method for the United States and Japan to establish a proof of concept for two very different cases of industrial development. Looking at a period of 75 years (1930–2005), we find that per capita MS has been much higher in the United States for the entire period, but that Japan has experienced much higher growth rates throughout, in line with Japan's late industrial development. By 2005, however, both Japan and the United States arrive at a very similar level of national MS of 310 to 375 tonnes per capita, respectively. This research provides new insight into the relationship between MS and flows in national economies and enables us to extend the debate about material efficiency from a narrow perspective of throughput to a broader perspective of stocks.

## Introduction

Two decades of research in the field of industrial ecology (IE) have created a rich knowledge base on the metabolic performance of nations, measured as material flows mobilized to fuel and sustain processes of production and consumption. This focus on “throughput” of materials has meant that the other dimension of society's materiality, namely, material stocks (MS), has been much less addressed in research. Yet, the amount of buildings, transport infrastructure, manufacturing capacity, and household appliances operated in a society have very close links to productivity and the material standard of living that can be achieved. MS and flows are in an intricate relationship. Stocks provide services and are established, fueled, and maintained through flows. The amount, age, and growth rate of stocks explain the demands for flows, both in terms of inputs and outputs. In IE research, MS play an important role in defining the focal system and its boundaries, which is a prerequisite for any analysis—once the system is defined in such a way, it becomes a straightforward task to identify relevant inflows and outflows. Nevertheless, the focus has been on material flows, rather than stocks, for numerous reasons. Material flows provide information about extraction rates from natural resource endowments and link to the issue of resource depletion. They are usually easy to establish at the national scale because of the large amount of economic data already available. On the other hand, the importance of measuring MS is much less clear for the policy community, and the measurement of stock quantities is more difficult and often relies on bottom-up approaches, which are time-consuming and data intensive.

Therefore, when available, nationwide stock accounts can typically be found as accompaniments for larger-scale statistics publications whose objectives do not focus on social metabolism, such as housing statistics (United Nations Economic Commission for Europe 2004), social capital (Cabinet Office of Japan 2007), and road and rail statistics (OECD 2012). Though usually comprehensive, these types of studies are restricted in scope and employ incompatible units of measurement (dwelling units and floor space, monetary value, and kilometers, respectively). This limits their practicality in IE research, unless time-consuming and elaborate manipulations are employed on the data. Statistical analysis of building stock lifespans has also been a topic of research, for example (Johnstone 1994), for housing stock in New Zealand and (Komatsu 2008) for buildings in Japan.

It is hence not surprising that national stock accounts are rare in the IE literature. The seminal study by the World Resources Institute (Matthews et al. 2000) is a rare example of measuring net additions to MS by comparing inputs to outputs within a very comprehensive material balance approach. The study presents data for five countries for the period 1975–1997, but provides no indication of total stocks for the countries analyzed. This approach has been utilized in other studies as well, for example, by the Ministry of the Environment of Japan (2009), which showed that net additions to stock decreased in Japan from 2001 to 2006.

MS accounting has instead focused on narrower scopes. Research has been limited either to specific materials, such as steel (Daigo et al. 2007; Allwood and Cullen 2012), iron (Mueller et al. 2006), copper (Gerst 2009), metals in general (Gordon et al. 2006), and, more recently, also carbon (Lauk et al. 2012) or limited in geographical scale to cities (Tanikawa and Imura 2001; Tanikawa and Hashimoto 2009) as a result of the intensive analysis required by the bottom-up methodologies used in those studies. Research has also been conducted into the relationship between flows and stocks (Mueller 2006; Hashimoto et al. 2007, 2009). Of these, Hashimoto and colleagues (2007) include an account of stocks in Japan for the years 1975–2030, but limited to minerals used in construction. Tanikawa and Imura (2000) presented accounts of the material stocked in selected types of infrastructure in Japan in the second half of the twentieth century, and the work was extended to 2005 in Nagaoka and colleagues (2009). Lichtensteiger and Baccini (2008) analyzed Switzerland's MS of buildings in the twentieth century.

This study aims to fill the gap in the literature by presenting a methodology for a top-down measurement of national MS and an application for two national economies, Japan and the United States. The study aims to

- establish national MS accounts from historical material flow accounts (proof of concept);
- analyze differences in economic efficiency of material throughput versus MS;
- explore possible courses of MS accumulation in the two countries using hypothetical future material flow scenarios; and
- investigate whether there is a saturation of MS of nations when an economy has matured.

In particular, the question of a saturation point in national MS has great relevance for the international debate on sustainable resource management. If such a saturation point exists and MS ceases growing at a certain point of economic development and maturity, then flows would potentially fuel and maintain this level of stock, preventing runaway throughput. This becomes an important issue for countries such as China, Brazil, and India, whose material use, at this point in time, dominates global material use.

Two countries were chosen for examination in this research: Japan and the United States. Both are highly developed countries boasting some of the strongest economies in the world today. On the other hand, their historical developments, as well as social, economic, and geographic characteristics, are quite different, offering an interesting comparison of material usage and accumulation.

## Methods and Data

### System Boundaries and Domestic Consumption Data

The spatial boundary of the examined countries is defined as the economies of countries, conforming with the Eurostat (2001) guidelines. Materials are considered as input flows into the system when they are either extractions from the domestic natural environment or imported from outside the geographical borders of the country into the economy. Likewise, output flows from the system are defined as either discharge of material back to the environment (as waste and emissions) or export from the country to the rest of the world. In accord with the data sources, the temporal boundary for this research is defined by the time unit of a year. A material that has both entered and exited the economy in less than a year will be considered throughput in the model, whereas material that remains in the system for longer than 1 year will be defined as stocked material.

This study employs long-term data compiled by Krausmann and colleagues (2011) for the years 1878–2005 for the inflow of materials for Japan and compiled by Gierlinger and Krausmann (2012) for the years 1870–2005 for the United States. These data sets follow the current standard methods and definitions of material flow accounting set out in Eurostat (2009). They contain yearly material flow statistics for approximately 60 material groups separated into imports, exports, and domestic extraction, whereas unused domestic extraction and indirect flows associated with imports and exports are excluded and, in any case, are not relevant because they do not enter the focal economy and hence cannot become part of the MS of that economy. In these data sets, the direct material consumption (DMC) of the economy in a year is given by equation (1) (Eurostat 2001):
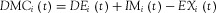
1where 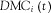 is the amount of domestic material consumption of material *i* in year *t*, 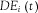 is the domestic extraction of material *i* in year *t*, 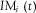 is the import of material *i* in year *t*, and 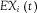 is the export of material *i* in year *t*.

For this study, material inflows related to the construction of buildings and civil infrastructure, the main portion of a national MS in terms of mass, were extracted from the two data sources. Construction materials not only make up the vast majority of MS on the national scale, but they also remain stocked in society for decades or longer, differentiating them from other major material categories, such as fossil energy carriers and biomass, which are used and discharged usually within much shorter time frames (Eurostat 2001).

The construction-related materials were grouped into four categories of timber, iron, other metals, and minerals to be used as inputs to the stock model. The other metals category includes metals other than iron, which are used in construction, such as copper, aluminum, and tin. The minerals category contains such nonmetallic materials as stone, sand, limestone, gravel, and clay. The stocking rates (the fraction of the DMC of these materials that becomes stocked) vary for each material group: DMC values contain a mix of raw and processed materials, semimanufactured materials, and final products, and the percentage of material that is used for construction and other types of consumption varies between material categories. Moreover, some materials that are used in the construction process of buildings and infrastructure, such as scaffolding, are either reused or become on-site demolition wastes, but do not form a part of completed structures and should not be accounted toward stocks. The actual amount of input material that becomes stocked (gross addition to stock, or GAS) is calculated with the following formulae shown in equations 2 and 3:
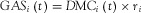
2a
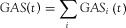
2bwhere 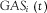 is the gross addition to stock, the new “layer” of addition to the total stock of material *i* in year *t*, 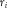 is the stocking rate of material *i* that is stocked as constructed structures in the anthroposphere, an aggregated percentage for which the assumed values are given in table1; and 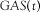 is the total gross addition to stock in year *t*.

**Table 1:** Assumed rate of input into stock of materials (r_i_) and variables for estimating the probability of survival of stocked material: Mean lifespan (μ_i_) and standard deviation (σ_i_)

*Material*	*Rate of input*	*Mean lifespan*,	*Standard*
*group (i)*	*into stock, % (r_i_)*	*years (μ_i_)*	*deviation (σ_i_)*
Timber	90	40	13.3
Iron	20	50	16.6
Other metals	10	50	16.6
Nonmetallic minerals	90	50	16.6

*Note:* Lifespan values adapted from Komatsu (2008) and Cabinet Office of Japan (2007).

### Lifespan Assumptions

There is very little information available regarding the time construction materials spend as stocks in the anthroposphere. To the best of the authors’ knowledge, no lifespan data for these materials as stocks are currently available, so lifespan probabilities of building and infrastructure lifespan in Japan (Komatsu 2008; Cabinet Office of Japan 2007) have been generalized and adapted for this research. This adaptation is based on the assumption that the lifespan of construction materials is similar to the lifespan of structures composed of them. The depreciation over time of materials that became stocked in a given year follows the s-shaped profile of the normal distribution's cumulative distribution function.

For the aims of the current study, some further generalizations and simplifications were made: Recycling processes are omitted because reliable, long-term recycling data are not available, and stocking rates and depreciation rates for construction materials are kept constant over time. Moreover, building lifespans in the United States are presumably longer than Japanese ones, because of historically lower building quality and fast material erosion of part of the Japanese housing stock, especially in single-family homes. Thus, the results were compared to existing research to assure reliability. Further, sensitivity analysis shows that different values for these variables have a relatively weak effect on the overall trends of MS accumulation over the study period and, certainly, no effect on the order of magnitude of the results. The statistical variables of stocking rates (*r_i_*) and survivability probabilities (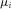 and 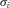) are provided in table1.

### Estimation of Material Stock

**Figure 1:**
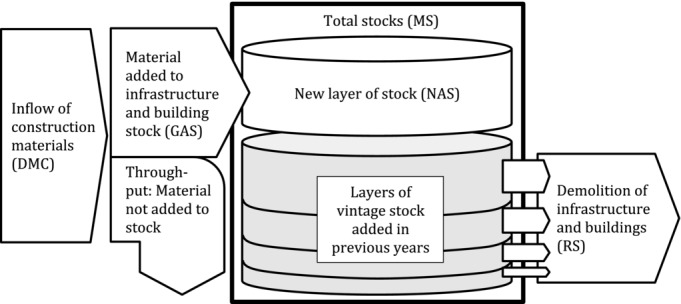
Framework of the model. DMC = direct material consumption; GAS = gross addition to stock; NAS = net addition to stock; MS = material stock; RS = removal from stock.

Simply stated, the approach taken here is to employ the aforementioned long-term material flow data sets for establishing MS accounts through a model of stock accumulation. The model framework is illustrated in figure 1. The four material categories are maintained throughout the metabolic processes of input, stock, and output. Each yearly input is considered a new layer of material added to the existing vintage stock from past years. Structures age and eventually get demolished and the quantity of material in each layer is reduced through a process of aging, estimated using the survival functions previously described in yearly calculation steps. The total stock of material in a given year is the sum of the material surviving (i.e., not yet demolished) from the layers of GAS from previous years, estimated with the following formulae shown in equations 4 and 5:
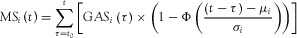
3a
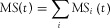
3bwhere 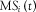 is the stock of material *i* in year *t*, *t*_0_ is the first year of DMC data (1878 for Japan and 1870 for the United States), and τ is the series of years from *t_0_* to the currently measured year *t*. In this summation function, τ also functions as the index of the yearly layers of previously stocked material; Φ is the cumulative distribution function of the standard normal distribution, providing the percent of remaining material *i* stocked in year *t* after 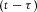 years have passed. 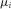 is the mean lifespan of material *i*, 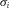 is the standard deviation of material *i* (the values of 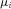 and 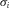 are given in table1), and 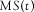 is the total MS in year *t*. Historical MS data do not exist for the periods preceding the material flow statistics (before 1870 and 1878 for the United States and Japan, respectively). We use the first year in the series until 1929 as a buffer for the accumulation of stock. According to our calculations, only 0.1% of the stock in 1930 is composed of material stocked in the first year of the series, suggesting that material stocked before that is negligible. This facilitates the selection of 1930 as the base year for this study.

### Removals from Stock

Eurostat (2001) defines net additions to stocks (NAS) as the GAS minus removals from stock (RS). NAS also describe the change in total stock from one year to the next, fundamentally being the differential of MS. This, together with a simple rearrangement of the Eurostat formula, reveals the material removed in a given year, as shown by equations 6 and 7:
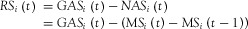
4a
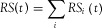
4bwhere *RS_i_*(*t*) is the RS of material *i* in year *t*, *NAS_i_*(*t*) is the NAS of material *i* in year *t*, and 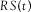 is the total material removed from stock in year *t*.

### Future Scenarios

The historical data sets for both Japan and the United States end in the year 2005. In addition to the historical analysis offered by the available data, the model was extended until the year 2050 using material flow projections. The main purpose of these projections is to further explore the mechanics of the model and present different scenarios of material usage and accumulation in the near future in search of the possibilities of reaching material saturation. As such, these projections should be considered “what if” proactive scenarios, not forecasts or predictions, such as those made with statistical regression methods.

Two scenarios of future MS accumulation with interesting profiles were selected. The scenarios are defined by differing GAS rates to be used as inputs in the period 2006–2050, whereas the model framework and assumed rates are unchanged from the historical period. In other words, the scenarios substitute equation 2, but equations 3 to (4) remain unaffected.

In the first scenario, material input (*GAS*) is kept at a steady rate based on the average of the material input of the final ten years of the data set (1996–2005) for this 45-year period, using equation 8:
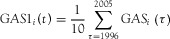
5where *GAS1_i_* is the amount of GAS of material *i* in year 2006 ≤ *t *≤ 2050 in scenario 1, and τ is the index of the final ten years of the historical series.

The second scenario couples changes to GAS to the projection of population growth, utilizing equation 6:
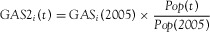
6where *GAS2_i_* is the amount of GAS of material *i* in year 2006 ≤ *t* ≤ 2050 in scenario 2, 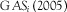 is the amount of GAS in the final year of the historical series, 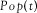 is the estimated population in year *t* using the sources described below, and *Pop*(2005) is the population in the final year of the historical series.

### Population and Gross Domestic Product Statistics

Data for population and gross domestic product (GDP) was used to determine per capita MS and material efficiencies. These statistics for Japan and the United States until 2005 were taken from Maddison (2008). Population statistics and projections for Japan from 2006 to 2050 are from the United Nations (2011) and for the United States from the US Census Bureau (2008).

## Results

### The Material Stock of Japan

**Figure 2:**
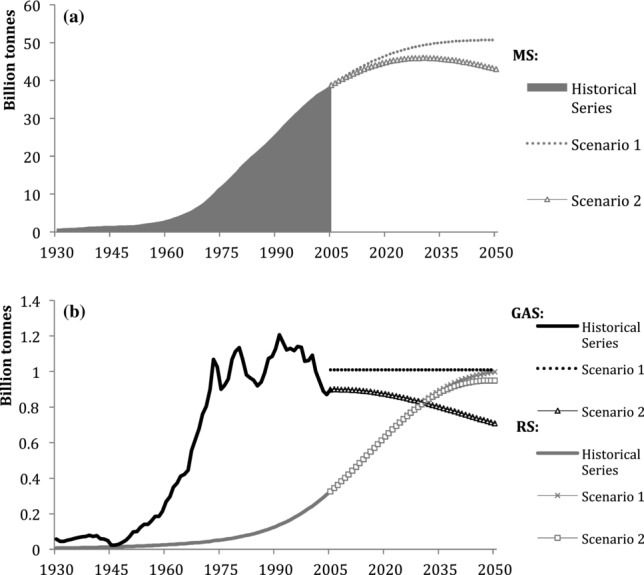
Stocks and flows in Japan, 1930–2050: (a) material stock (MS), (b) gross additions to stock (GAS), and removals from stock (RS).

Our modeling results show, for a period of 75 years (1930–2005), that the total stock of construction materials 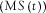 in Japan grew by a factor of 40, from approximately 920 million tonnes in 1930 to 38.7 billion tonnes in 2005 (figure 2a). The trend is characterized by slow growth in the prewar period up to the 1950s, during which MS doubled. It should be noted that singularities of MS loss, such as the destruction of substantial amounts of Japanese infrastructure in World War II, are unobservable as a result of the nature of the model. The postwar period is characterized by an immense increase in MS driven by rapid economic growth and the modernization and urbanization of Japan. By the early 1970s, GAS had reached approximately 1 billion tonnes per year and remained at that rate until the end of the twentieth century. In recent years, fluctuating inflows and ongoing increases of outflows of material stocked in previous decades (figure 2b) have led to an apparent slowdown in MS growth. The future scenarios both point toward visible slowdown in MS growth. Assuming a future inflow of construction materials similar in quantity to the average of the last decade (scenario 1), overall stocks will continue to increase, albeit at slower and slower rates, reaching 50 billion tonnes by 2050 and implying an s-shaped growth curve for MS, perhaps leading toward a saturation of total stocks at some point in the future. The second scenario is linked to Japan's population, which is projected to decrease dramatically (United Nations 2011), in effect reducing the GAS to such low levels that it will become less than RS by 2031 and leading not only to a saturation of MS, but also to dematerialization. Consequently, in 2031, MS will reach a peak of 45.9 billion tonnes and stocks will reduce afterward, diminishing to only 43 billion tonnes in 2050. This is approximately 15% less than the MS of scenario 1 in 2050. It is notable that the trend of RS is constantly increasing, reaching almost 1 billion tonnes per year by 2050 and is hardly affected by the premises of the two scenarios, because most of the removed material originated in the historical additions to stock.

**Figure 3:**
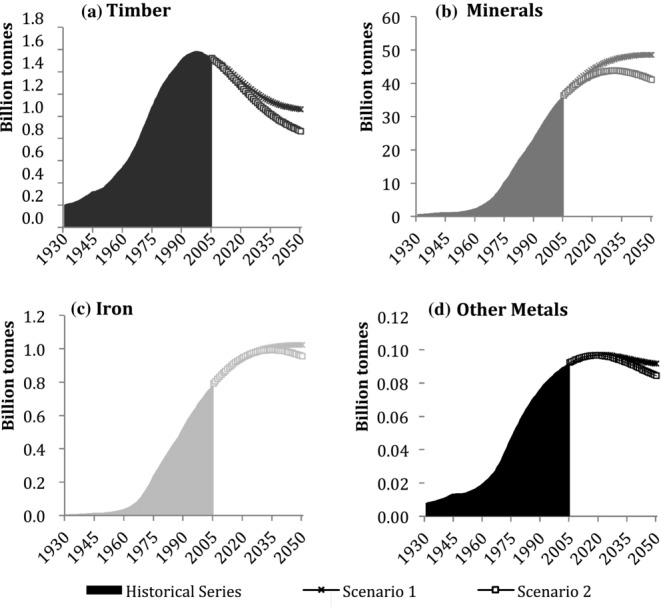
Individual stocks trends of materials in Japan, 1930–2030: (a) timber, (b) minerals, (c) iron, and (d) other metals. Note the different scales.

Figure3 shows the four stock categories, which have evolved differently, mirroring the changes in preferred construction materials and technologies over the twentieth century in Japan. Iron, other metals, and minerals stocks have been rapidly increasing since the beginning of the 1960s in conjunction with Japan's economic growth phase. Iron stocks in 2005 were 791 million tonnes, 109 times the stock quantities of 1930, showing the largest relative increase of the four examined categories. Construction minerals stock has grown by a factor of 66 from 703 million tonnes in 1930 to 36 billion tonnes in 2005, whereas the stock of other metals has only grown approximately 12-fold, from almost 8 million tonnes in 1930 to more than 92 million tonnes in 2005.

Construction minerals and iron and steel stocks are continuing to grow, albeit at slower rates. The stock of other metals shows saturation during the next couple of decades, and timber stocks peaked around 1995 at 1.3 billion tonnes and have since been in decline.

Both scenarios project further decreases to timber, and other metals are projected to peak in the 2020s. Minerals and iron stocks would stabilize in the first scenario, but peak and then decline in the second one.

Throughout the study period, the major constituent group of Japan's MS was minerals. Their share rose from 76% in 1930 to 95% in 2005 and is expected to rise to 96% by 2030. In comparison, timber made up 22% of Japan's MS in 1930, but declined to approximately 3% in 2005. It is interesting to note that in the immediate postwar period, timber regained in importance to a share of 18%, but since then has constantly declined and is projected to reach 2% by 2050. The share of other metal stock, including materials such as copper and aluminum, was small during the entire study period. Iron had increased from 1% to 2% by 1964 and has remained at that rate since. In both future scenarios, these percentages do not change significantly until 2050.

### The Material Stock of the United States

**Figure 4:**
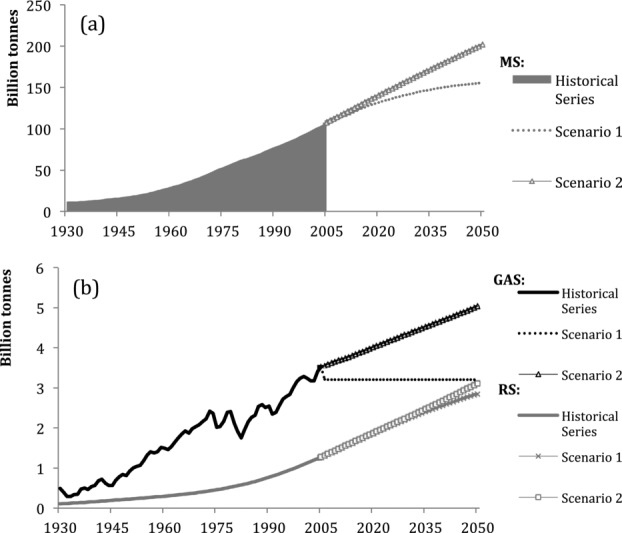
Stocks and flows in the United States, 1930–2050: (a) material stock (MS), (b) gross additions to stock (GAS), and removals from stock (RS).

Figure4a shows that stocks of construction materials in the United States were higher than in Japan during the entire study period, reflecting a larger, more populous economy and a higher level of wealth. Total stocks grew from 11 billion tonnes in 1930 to 107.5 billion tonnes in 2005, a ninefold increase. The growth trend was characterized by steadily increasing amounts of yearly additions to stock throughout, despite some fluctuations in the 1970s. RS have been slowly increasing as well (figure 4b), at lower levels than inflows. As a result, MS in the United States continued to increase until 2005. Our model suggests that, in the first scenario, the rate of accumulation of MS will decrease and MS will reach 155 billion tonnes, but the second scenario points toward continuously increasing stocks caused by the projected further growth of population in the United States until 2050. In the United States, this scenario behaves in an opposite way from the Japanese case. RS reach almost 3 billion tonnes per year in 2050 in both scenarios.

**Figure 5:**
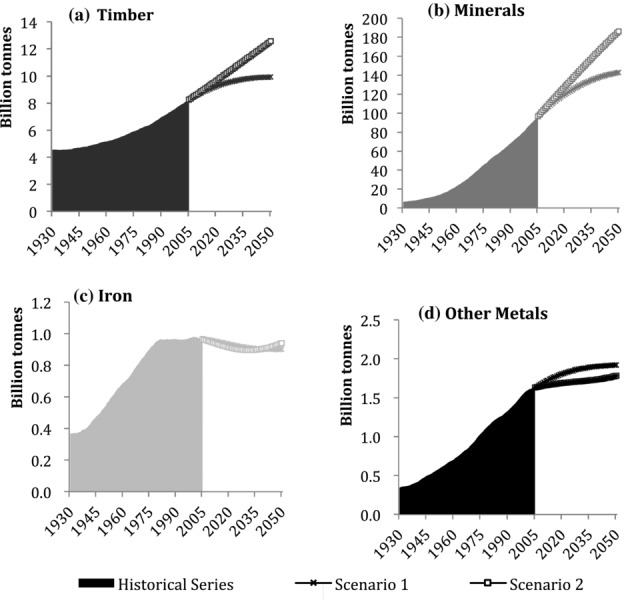
Individual stocks trends of materials in the United States, 1930–2030: (a) timber, (b) minerals, (c) iron, and (d) other metals. Note the different scales.

The trends of each material category are shown in figure 5. Construction mineral stock was 6.5 billion tonnes in 1930 and 96 billion tonnes in 2005, a 15-fold increase that was characterized by a moderate growth until the beginning of the 1950s, after which a sustained rapid growth is ongoing. Timber has followed a similar growth curve, but much more restrained, almost doubling from 4.5 billion tonnes in 1930 to 8.2 billion tonnes in 2005. Iron stock increased from 366 million tonnes in 1930 to 950 million tonnes in 1979, and since then, its growth has almost halted, reaching approximately 970 million tonnes in 2005. Other metals show no similar slowdown. This category's stock grew from 346 million tonnes to 1.6 billion tonnes in 2005. Timber and mineral stocks are expected to continue growing in both scenarios, whereas the growth of iron and other metals stocks seems to come to an end in both cases. Similar to Japan, the major component of MS is nonmetallic minerals. These materials had a share of 55% of total stock in 1930 and increased to 90% in 2005. Concrete gradually replaced timber as a major construction material, and timber lost in share from 39% in 1930 to less than 10% by the 1980s. At the end of the 1960s, the succession of timber by construction minerals was complete and the composition of stock has not changed significantly since. Similar to Japan, both iron stocks and other metal stocks had only a small and decreasing share of total stocks in the United States, from 3% to 1% each, despite growth in absolutest numbers.

The results show that, by 2005, MS in both Japan and the United States continued to increase and are not saturated yet. On the contrary, total stocks in both countries, fueled mainly by minerals accumulation in buildings and infrastructure, have not shown any significant slowdown. However, future scenarios of Japan offer projections of total stock saturation and even dematerialization. In the United States, even though no scenario presents such a trend, scenario 1 does present a slowdown of MS growth, suggesting that, at some point in the future, stabilization of stocks could occur there as well.

## Discussion

### Validity of the Model and Sensitivity Analysis

Previous research (Hashimoto et al. 2007) calculated the construction-related MS growth of Japan for the years 1976–2005 using a bottom-up method of stocking rates of materials per unit of floor space for several types of buildings and material intensities for different infrastructure and provided a projection of growth until 2030. The materials examined in the research were limited to asphalt, cement, sand, gravel, and crushed stone, which correspond with materials in the category of construction minerals in our research. Nagaoka and colleagues (2009) employed a method of material intensities per unit of construction and focused on the materials found in buildings, roads, and sewerage systems for the period 1945–2005.

Because of a more limited scope of these previous studies (fewer materials in Hashimoto et al. 2007 and fewer construction types in Nagaoka et al. 2009), we would expect our results to be higher than those presented in previous studies.

**Figure 6:**
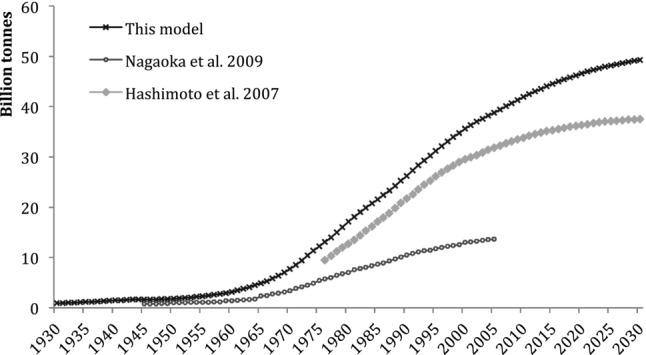
Comparison between construction material stock estimations for Japan: The studies published by Hashimoto and colleagues (2007) for 1976–2030, Nagaoka and colleagues (2009) for 1945–2005, and this research, scenario 1, for 1930–2030.

A year-by-year comparison (figure 6) shows that the figures for Japan's MS (Nagaoka et al. 2009) are, on average, 40% of our estimate because some of the most material-intensive infrastructure projects, such as harbors, airports, in agriculture, and landslide/flood control, had not been accounted for in that research. The aggregate numbers (Hashimoto et al. 2007) are very similar to our estimates. Hashimoto and colleagues estimated material stocks in Japan at approximately 20% lower than our estimate, mainly the result of a more limited scope of material categories. The low variance in the year-by-year difference between our results and the previous studies shows very similar growth trends of all three studies.

**Figure 7:**
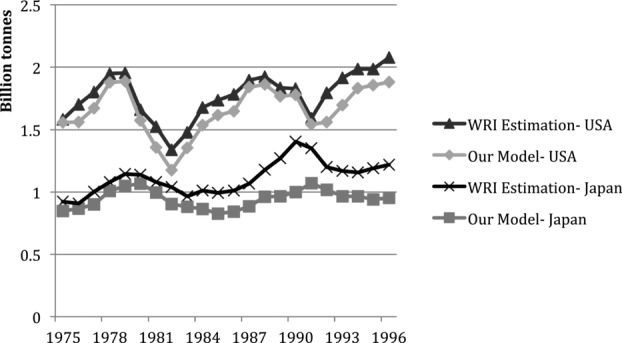
A comparison between net additions to stocks (NAS) estimations for Japan, 1975–1996: the study published by the World Resources Institute (Matthews et al. 2000) and this research.

Although there are no MS studies of the United States for comparison, figure 7 shows that the values and trends of change in net additions to stocks obtained by Matthews and colleagues (2000) for both the United States and Japan are very close to the results of the current research, despite the different methodologies employed. The scope of Matthews and colleagues contains materials not related to construction, which only contribute small amounts to the additions to stock and which are not included in this study. Accounting for these materials in the previous study explains their slightly larger results.

The comparison with previous studies shows a high coincidence in both level and evolution of national MS and makes us confident about the validity of our analytical approach.

Sensitivity analysis was conducted on the model to understand the effects of changes in stocking rates and lifespan assumptions. Because the stock account is derived from a model, sensitivity analysis is a straightforward task. By definition of the model, increasing or decreasing stocking rates changes the resulting stock account by the exact same proportion. Lifespan variables have a weak effect on overall stock. A 10% change in mean lifespan results in a 2% change in the stock account, on average. These variables have relatively weak effects because the aggregation of the dozens of layers of stocks has a dampening effect on changes in lifespans. The modeling approach, in contrast to a bottom-up analysis, also allows for testing assumptions about future trends in stock, amounts of input required, and amount of materials that will be discarded. This may well become policy-relevant information if economic planning would focus on extending the durability and lifetime of buildings and infrastructure to allow a shift from a throughput metabolic pattern to a focus on stocks and services derived from the stock.

### Material Stocks per Capita

**Figure 8:**
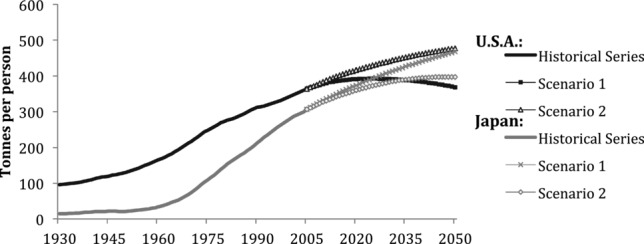
Material stocks per person in the United States and Japan, 1930–2050. Source: own calculations using population data from Maddison (2008), United Nations (2011), and US Census Bureau (2012).

Figure8 compares the level and evolution of per capita material stock in Japan and the United States. The United States started at a much higher level of MS of approximately 100 tonnes per capita in 1930, reflecting its higher level of industrialization and wealth. MS in Japan was only one fifth of that in the United States at that time. Japan has since shown a much higher dynamic of MS growth, increasing 20-fold, whereas MS in the United States grew fourfold. Despite much faster growth in Japan, the United States was still ahead of Japan in per capita MS in 2005, with 375 tonnes per capita, compared to 310 tonnes per capita in Japan. It appears that despite the different historical developments and social and economic differences, a certain level of MS per person are to be expected in developed countries with mature economies. Rubli and Jungbluth (2005) quantified MS for Switzerland at 311 tonnes per capita, which appears to be in line with our results. However, Japan's population projections are negative (United Nations 2011) and the United States projections are positive (US Census Bureau 2008). The per person stocks in these countries are projected to change in quite different ways in each of the future scenarios. In scenario 1, by 2050, stocks in Japan will increase to 467 tonnes per capita, whereas the United States’ per person stocks will peak at 392 tonnes per capita and then decline to 368 tonnes per capita. Japan's per capita MS levels will surpass the United States by 2023.

On the other hand, the dynamics of scenario 2 offer another course in which the stock per person in the United States remains higher than in Japan. In this scenario, stocks in the United States continue to grow to 476 tonnes per person in 2050, whereas in Japan, stocks per person will stabilize at aproximately 395 tonnes per person by 2042.

Steady or decreasing per capita stock could be termed per capita material saturation. In the case of the United States, this can be achieved using policies that reflect scenario 1, which, in fact, lead to per capita dematerialization, but in Japan, scenario 1 has opposite results—the materials per person will continue to increase despite reaching toward saturation in absolute terms. From these, it can be understood that pursuing policies of a certain scenario in one country would not necessarily provide the same results in another.

### Material Efficiency of Japan and the United States

One important measure for the success of sustainable resource management at the national economy level is material efficiency—the amount of economic output per unit of material use. Most studies show that economic material efficiency increases as economies mature (Schandl and West 2010), which is caused by a growing share of minerals and fossil fuels and declining share of biomass and the different income elasticities of those different materials (Steinberger and Krausmann 2011). These previous studies discuss only material throughput, and the model presented here allows a long-term comparison with stock efficiency as well. Because stocks provide services to society, more-effective usage of existing stocks is of benefit to both the economy and the environment. Nevertheless, material flow efficiency is an indicator of the flows and economic activity of a certain year, whereas MS efficiency measures the efficiency of the accumulation of materials over time. Therefore, an appropriate comparison between these two indicators is to look at the trends over time and not a year-by-year comparison.

**Figure 9:**
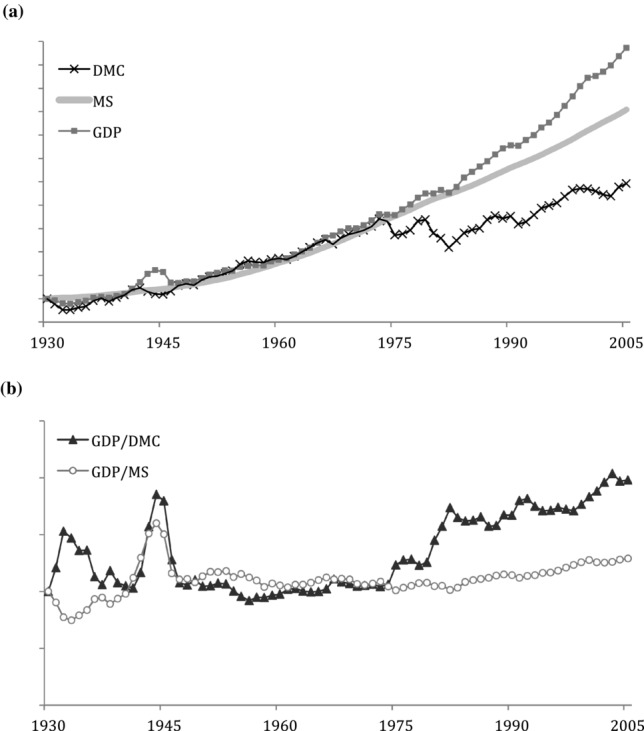
Comparison of the growth rates in the United States, 1930–2005. Values normalized to 1930 = 1: (a) direct material consumption (DMC), material stock (MS), and gross domestic production (GDP); (b) material productivity of material input (GDP/DMC) and material productivity of material stock (GDP/MS). GDP statistics from Maddison (2008).

In the United States, we see a trend until about 1975 with all three individual indicators (DMC, MS, and GDP) growing in concert (figure 9a). Delinking started from 1974, when GDP growth rates outpaced growth in both MS and flows. This increased efficiency is visualized in figure 9b, which shows both material efficiency trends of the United States. The material efficiency measures were moving in concert from World War II until 1974, and since then, material flow efficiency, measured as GDP/DMC, is increasing, slowly being trailed by improvements in MS efficiency when measured as GDP/MS.

**Figure 10:**
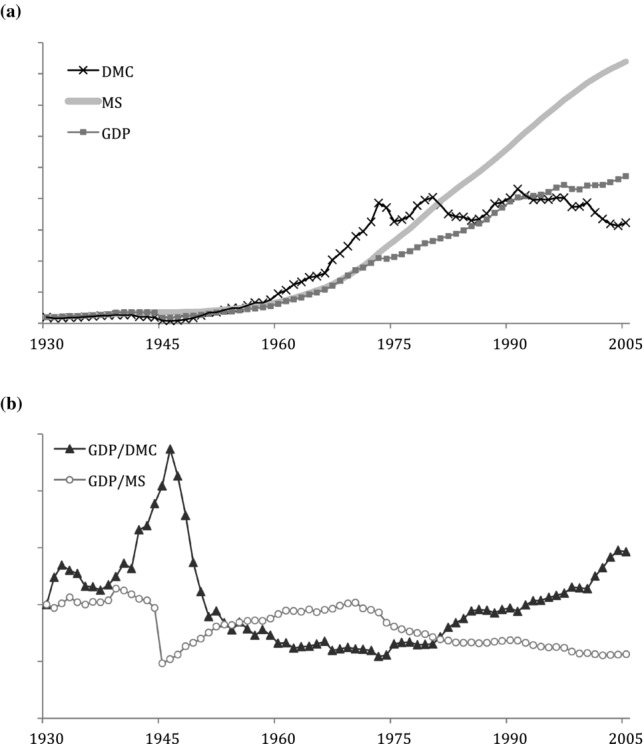
Comparison of the growth rates in Japan, 1930–2005. Values normalized to 1930 = 1: (a) direct material consumption (DMC), material stock (MS), and gross domestic production (GDP); (b) material productivity of material input (GDP/DMC) and material productivity of material stock (GDP/MS). GDP statistics from Maddison (2008).

Figure10a shows growth rates for DMC, GDP, and MS for Japan. All three indicators evolved on a similar trajectory from the 1930s until 1975. Since 1975, DMC has stabilized and MS have grown much faster than GDP. Measuring material efficiency of the Japanese economy based on both material indicators (DMC and MS) shows an inverse trend for the two (figure 10b). As mentioned in the “Results,” singularities such as the effects of World War II on Japanese MS are omitted in this type of model and deserve more detailed study. This is obvious during the 1940s, where we observe extreme divergent trends for the two efficiency measures. From the early 1950s until about 1972, the material efficiency of stock (GDP/MS) was increasing whereas the material efficiency of throughput (GDP/DMC) was decreasing. This was a period of fast-growing material input to fuel the economic growth period of Japan. Subsequent to that, a new trend has been triggered, probably by increasing oil prices and a genuine slowdown in the Japanese economy, which has meant a turning point for material efficiency. Flow efficiency has increased since then, making Japan the most material-efficient economy globally (Schandl and West 2012). Because stock has continued to grow beyond GDP growth rates, a material efficiency measure based on MS shows declining or stagnant productivity. The amount of MS needed for economic activity has not declined at all, despite a long period of deinvestment in Japan since the 1990s (Randers 2012). This raises the question of the reasons for the ongoing increases in MS and several answers are possible. First, Japanese building standards are periodically updated to enforce increasingly more-intensive construction to withstand Japan's susceptibility to natural disasters, such as earthquakes (Tanikawa and Hashimoto 2009), requiring more material per unit in each new or replaced structure. A second reason is the growing affluence of Japanese society, creating demand for bigger and higher-quality construction. Third, nationwide infrastructure projects, such as high-speed rail, dams, and national highways, have been constantly expanding through government policy. Japan's rugged mountainous terrain necessitates vast numbers of bridges, tunnels, and other material-intensive civil engineering structures to support these kinds of projects, which contribute to the increase in stocks. A fourth reason could be that some of the stock included in this calculation is actually dissipated stock, such as disused buildings and infrastructure, which still “hibernate” in the anthroposphere, but make no meaningful contribution to the economy (Brunner 2004; Hashimoto et al. 2009). Unfortunately, the lack of data on abandoned infrastructure for Japan or any other country hinders answering this question at this time.

## Conclusions

In this study, we developed a novel method for a top-down account of MS of national economies based on historical material flow studies and applied the approach for two national economies, the United States and Japan. We were able to prove that the approach delivers credible results and has merits, in comparison to bottom-up approaches, for stock accounts, which are usually very time-consuming and costly. We compared the evolution of national MS for the two case studies and showed that, by 2005, both economies reached comparable levels of per capita material stock of approximately 310 tonnes per person in Japan and 375 tonnes per person in the United States. These results show that material saturation has not been reached in either of these countries, because the growth of MS, both per capita and in absolute numbers, has been continuous until 2005. We presented two scenarios until 2050, which suggest possibilities of reaching saturation in the future and even dematerialization of total stocks in Japan and dematerialization of per capita stocks in the United States. Despite the relatively simplified scenario assumptions, the scenarios may be useful for policy making, as well as for the management of future material requirements for stock maintenance and the expected amounts of demolition waste. Nevertheless, because this approach is input driven, it is unable to show mass output of construction MS events, such as the destruction of assets that occurred during World War II, Japan's 2011 Tsunami, or the United States’ 2005 Hurricane Katrina.

We also compared the trends of material efficiency of throughput to material efficiency of stock and saw that, in the United States, both efficiencies have improved since the 1970s, albeit the efficiency of stock much less so. In Japan, we saw the improving material efficiency of throughput that goes hand in hand with declining efficiency of MS caused by fast rates of growth. This trend in Japan is further exacerbated by the fact that the share of throughput that goes to stock is much larger than in the United States, which employs a throughput metabolic pattern (Matthews et al. 2000). This new information on national MS may become very useful in an economic context that refocuses on the services delivered by quality buildings and infrastructure, based on a new focus of lowering throughput of materials and achieving a steady state.

A characteristic that is absent in the current discussion is the geosocial aspect of MS. Buildings and infrastructure are, by their nature, fixed assets, taking up physical space on land and altering its shape. The total area taken up by MS, the spatial distribution of MS, and the available land for potential construction all affect MS accumulation values and trends. Countries have unique land areas, land use, building densities, and other geosocial differences. We examined the saturation of material in both absolute terms and per person, but further country comparisons and material saturation could be potentially examined through the lens of geography in addition to the economic and population perspectives presented thus far. Moreover, trends of material efficiency in Japan and the United States would warrant additional analysis. In a comparison of the conjoint behavior of flow efficiency and stock efficiency in the two countries, some correspondence becomes visible. For example, flow efficiency increased and stock efficiency decreased during both countries’ economic recessions: the United States’ great depression (1930s) and Japan's “lost decade” (1990s). Whether these, and other such similarities, are coincidental or characteristic of material-economic dynamics should also be subject to further study.

## About the Authors

**Tomer Fishman** is a research fellow at the Graduate School of Environmental Studies of Nagoya University, Nagoya, Japan. **Heinz** Schandl is a senior principal scientist at the Commonwealth Scientific and Industrial Research Organization (CSIRO) Ecosystems Sciences division based in Canberra, Australia, an adjunct associate professor at the Australian National University (ANU) School of Sociology, and visiting professor at the Graduate School of Environmental Studies of Nagoya University. **Hiroki Tanikawa** is professor of environmental systems research at the Graduate School of Environmental Studies of Nagoya University. **Paul Walker** is an honorary research fellow with CSIRO Ecosystem Sciences. **Fridolin Krausmann** is professor of sustainable resource use at the Institute of Social Ecology at Alpen Adria University, Vienna, Austria.
